# Epicuticular wax on cherry laurel (*Prunus laurocerasus*) leaves does not constitute the cuticular transpiration barrier

**DOI:** 10.1007/s00425-015-2397-y

**Published:** 2015-09-04

**Authors:** Viktoria Zeisler, Lukas Schreiber

**Affiliations:** Department of Ecophysiology, Institute of Cellular and Molecular Botany, University of Bonn, Kirschallee 1, 53115 Bonn, Germany

**Keywords:** Cuticular transpiration, Cuticular wax, Leaf surface, Plant cuticle, Wax chemistry

## Abstract

**Epicuticular wax of cherry laurel does not contribute to the formation of the cuticular transpiration barrier, which must be established by intracuticular wax.**

Barrier properties of cuticles are established by cuticular wax deposited on the outer surface of the cuticle (epicuticular wax) and in the cutin polymer (intracuticular wax). It is still an open question to what extent epi- and/or intracuticular waxes contribute to the formation of the transpiration barrier. Epicuticular wax was mechanically removed from the surfaces of isolated cuticles and intact leaf disks of cherry laurel (*Prunus laurocerasus* L.) by stripping with different polymers (collodion, cellulose acetate and gum arabic). Scanning electron microscopy showed that two consecutive treatments with all three polymers were sufficient to completely remove epicuticular wax since wax platelets disappeared and cuticle surfaces appeared smooth. Waxes in consecutive polymer strips and wax remaining in the cuticle after treatment with the polymers were determined by gas chromatography. This confirmed that two treatments of the polymers were sufficient for selectively removing epicuticular wax. Water permeability of isolated cuticles and cuticles covering intact leaf disks was measured using ^3^H-labelled water before and after selectively removing epicuticular wax. Cellulose acetate and its solvent acetone led to a significant increase of cuticular permeability, indicating that the organic solvent acetone affected the cuticular transpiration barrier. However, permeability did not change after two subsequent treatments with collodion and gum arabic or after treatment with the corresponding solvents (diethyl ether:ethanol or water). Thus, in the case of *P. laurocerasus* the epicuticular wax does not significantly contribute to the formation of the cuticular transpiration barrier, which evidently must be established by the intracuticular wax.

## Introduction

Leaves and fruits are covered by an extracellular polymer membrane, the plant cuticle, which limits the transpirational water loss (Schreiber and Schönherr 2009), reflects UV-radiation (Krauss et al. 1997), protects leaves from infection by pathogens (Riederer and Müller 2006) and provides a self-cleaning mechanism known as the Lotus effect (Neinhuis and Barthlott 1997). The cuticle is composed of the insoluble polymer cutin and soluble cuticular lipids commonly called wax. Cutin is a biopolyester mainly consisting of C_16_ and C_18_ hydroxy-fatty acids (Pollard et al. 2008). These fatty acids are cross-linked by ester bonds, creating the mechanically stable cutin polymer network (Espelie et al. 1980; Kolattukudy 1981; Nawrath 2006).

Cuticular wax, which is synthesised in epidermal cells, is composed of a mixture of linear, long-chain aliphatic molecules of different functionalities and different chain lengths. In some species it also contains significant amounts of pentacyclic triterpenoids (Samuels et al. 2008; Kunst and Samuels 2009). Cuticular wax is embedded in the cutin polymer (intracuticular wax) and deposited on the outer surface of the cuticle (epicuticular wax) (Buschhaus and Jetter 2011), where it can form characteristic, three-dimensional epicuticular wax crystalloids (Jeffree 1986; Barthlott et al. 1998). It is well established that the cuticular transport barrier for water and solutes is established by cuticular wax, since upon wax extraction cuticular permeability increases by two to three orders of magnitude (Schönherr 1976; Schönherr and Riederer 1989; Schreiber and Schönherr 2009). It is still unknown to what extent epi- and/or intracuticular waxes contribute to the formation of the cuticular transpiration barrier.

It has been shown that various environmental factors (e.g. light, drought, high temperature) can lead to an increase in the amounts of epicuticular wax deposits and changes in their appearance (Grant et al. 1995; Sase et al. 1998; Kim et al. 2007), thus reducing absorption of heat by reflection of sun light. From these microscopic observations, it is often concluded that an accumulation of epicuticular wax in response to environmental stress should also reduce rates of cuticular transpiration. Up to now, convincing experimental evidence confirming or rejecting this suggestion is largely missing. This is due to the fact that adequate experimental approaches have been limited.

Within the last few years new techniques for studying the chemical composition of epicuticular wax separately from intracuticular wax have been developed. Different approaches allowing the mechanical removal of epicuticular wax separately from intracuticular wax have been described (Haas and Rentschler 1984; Ensikat et al. 2000; Jetter et al. 2000; Koch et al. 2004). Jetter et al. (2000) demonstrated for the first time that cuticular wax of *Prunus laurocerasus* is arranged in chemically distinct layers. Pentacyclic triterpenoids were shown to be nearly exclusively located within the cutin polymer, whereas linear, long-chain aliphatic molecules occurred in both the epi- and intracuticular wax fractions (Jetter et al. 2000; Jetter and Schäffer 2001). In the following years the observation that cuticular wax is arranged in distinct layers with the epicuticular wax fraction being chemically different from the intracuticular wax fraction was confirmed for other species (Buschhaus and Jetter 2011). It has frequently been observed that triterpenoids are more abundant or dominant in the intracuticular wax fraction, whereas linear, long-chain aliphatic molecules are present in more equal amounts in both wax fractions (Buschhaus and Jetter 2011).

This highly organised and layered structure of cuticular wax prompts the question whether this arrangement has any function? When investigating plant/pathogen interaction and host recognition, it was shown that specific chemical cues in the outermost epicuticular wax fraction are essential for plant/pathogen recognition (Ringelmann et al. 2009; Hansjakob et al. 2010). This indicated for the first time that epicuticular wax is not only essential for the interaction of plants with the abiotic environment (Grant et al. 1995; Sase et al. 1998; Kim et al. 2007) but with the biotic environment as well. Ultrastructural studies, using atomic force microscopy and looking at the regeneration of epicuticular wax in vivo after its selective removal, indicated that regeneration of epicuticular wax can start within minutes (Koch et al. 2004).

In view of these different approaches for studying the function of epicuticular wax for plant/environment interactions, it is evident that detailed and comprehensive studies investigating the role of epicuticular wax in determining cuticular transport properties, especially cuticular transpiration, are largely missing. To our knowledge there are only a few published studies that have measured cuticular transpiration before and after removal of epicuticular wax. Results lead to very different conclusions. For barley wax mutants it was concluded that “cuticular transpiration is poorly correlated to the amount or composition of the epicuticular lipids” (Larsson and Svenningsson 1986). Baur (1998) observed no effect at all with intact leaves and isolated cuticular membranes of different species upon removal of epicuticular wax, whereas in two other studies, looking at fruit cuticles of either tomato (Vogg et al. 2004) or cherry (Knoche et al. 2000), a two to three fold increase of cuticular transpiration was observed after removal of epicuticular wax. Keeping in mind that cuticular permeability for water and solutes increases by two to three orders of magnitude upon total wax extraction (Schönherr 1976), this is not a very pronounced effect.

As it is currently not possible to answer clearly the question to what extent epicuticular wax contributes to the formation of the cuticular transpiration barrier, we investigated water permeability of isolated cuticles and intact leaf disks of *P.laurocerasus* before and after mechanical removal of epicuticular wax. *P. laurocerasus* was chosen because wax composition (Jetter et al. 2000; Jetter and Schäffer 2001) and cuticular transport properties (Kirsch et al. 1997; Schreiber et al. 2001) have been intensively studied in the past. Wax removal was carried out in our study with three different methods, using collodion (Haas and Rentschler 1984), cellulose acetate (Silcox and Holloway 1986) and gum arabic (Jetter and Schäffer 2001). Collodion and cellulose acetate were chosen because a rapid technique was needed in view of the observation that wax recrystallization after mechanical wax removal can occur within minutes (Koch et al. 2004). Gum arabic was chosen as a third agent for the selective removal because it is dissolved in water and not in an organic solvent, which could potentially dissolve intracuticular waxes and thus disturb the transpiration barrier. Three different techniques were applied to elucidate structure–function relationships of epicuticular wax: (1) gas chromatography and mass spectrometry for analyzing wax composition in the different wax fractions obtained by the polymer treatments; (2) scanning electron microscopy for investigating the cuticle and leaf surface before and after mechanical removal of epicuticular wax; (3) measurement of cuticular water permeability using radiolabelled water, allowing measurement of cuticular transpiration with the highest accuracy and quickly (within minutes). This combined experimental approach allowed us to conclude that epicuticular wax of *P. laurocerasus* does not significantly contribute to the formation of the cuticular transpiration barrier, which must obviously be formed by intracuticular wax.

## Materials and methods

### Plant materials

Leaves of *Prunus laurocerasus* were harvested from plants growing in a rural area outside of Bonn. Leaf disks (LDs) were punched out from freshly harvested leaves with a cork borer (2 cm diameter), avoiding major leaf veins. In some experiments, transpiration across the upper (adaxial), astomatous side of the LDs was investigated directly and cuticular membranes (CMs) were isolated for further experiments afterwards. In a parallel experiment, CMs from the upper, astomatous leaf side were isolated before measuring transpiration. For isolation of the CM, LDs were incubated in an enzymatic solution containing 2 % cellulose (Novozymes) and 2 % pectinase (Novozymes) dissolved in 0.01 M citric buffer (Roth) with the pH adjusted to 3.0 (Schönherr and Riederer 1986). In order to prevent microbial growth the enzymatic solution contained 1 mM sodium azide. After a couple of days, the astomatous upper CMs became detached from the remaining leaf disks and could be separated from the remaining leaf material. CMs were kept in fresh enzymatic solution for another few days, then transferred to borate buffer (Roth) adjusted to pH 9.0. Finally CMs were carefully washed with deionised water, air-dried using a gentle stream of nitrogen and stored in Petri dishes at room temperature for 2–3 months, until used in the experiments.

### Mechanical removal of epicuticular wax from CMs using collodion

**Fig. 1:**
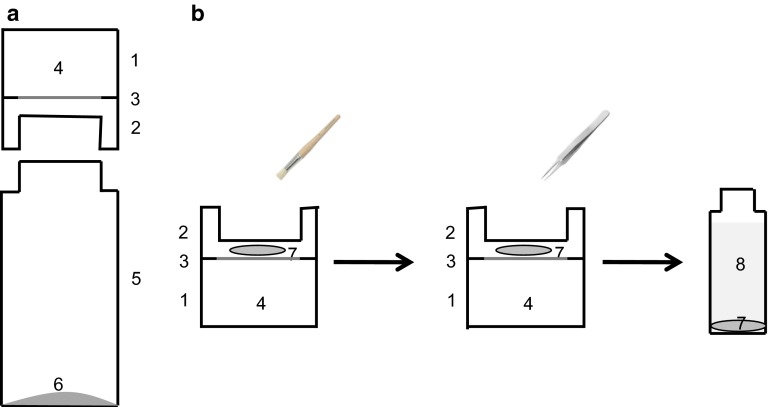
Schematic drawing of the experimental setup for measuring cuticular transpiration of *P. laurocersus* cuticles (CMs) and leaf disks (LDs) before and after treatment with collodion, cellulose acetate, gum arabic or the respective solvents. **a** CMs (3) or LDs (3) mounted between a stainless steel transpiration chamber (*1*) and an adapter (*2*), with the outer side of the cuticle or the upper side of the leaf facing the atmosphere, fixed upside down to a scintillation vial (*5*). ^3^H-labelled water (*4*) diffusing across the cuticle is quantitatively trapped in glycerol (250 µl) at the bottom of the scintillation vial (*6*). **b** For mechanical removal of epicuticular wax, the outer surfaces of CMs and LDs were treated twice with a drop (about 15 µl) of collodion, cellulose acetate or gum arabic (*7*) applied with a soft brush. After evaporation of the solvent the polymerised film was lifted off with tweezers

Epicuticular wax was removed from CMs of *P. laurocerasus* with collodion (Fluka). This approach was first suggested by Haas and Rentschler (1984). Collodion, which is nitrocellulose (4–8 %) dissolved in diethyl ether:ethanol (1:1, v/v), was applied gently (about 15 µl) with a soft brush on the outer surface of CMs (Fig. 1b). A thin, dry polymer film appeared after 30–60 s as the solvent evaporated. The polymerised nitrocellulose, with the epicuticular wax adhering to it, was carefully removed with fine tweezers. One and the same CM could be treated consecutively with collodion for up to five times without showing visible damage. For wax extraction and further analytical investigations, the collected polymer films were dissolved individually in 4 ml chloroform (Carl Roth). This is a very good solvent for cuticular wax extraction (Riederer and Schneider 1989), but it does not dissolve nitrocellulose.

### Mechanical removal of epicuticular wax from CMs using cellulose acetate

Cellulose acetate (Sigma-Aldrich) dissolved in acetone (5 %) was used in the same way as collodion for removal of epicuticular wax. Originally, Silcox and Holloway (1986) described this method for quantitatively collecting the residue of agrochemicals from leaf surfaces. Cellulose acetate (about 15 µl) was applied gently onto the surface of isolated *P. laurocerasus* CMs using a brush (Fig. 1b). After evaporation of the organic solvent (1–2 min) a cellulose acetate strip could be easily removed from the surface and extracted in 4 ml chloroform (Carl Roth) for analytical purposes. One and the same CM could be treated consecutively with cellulose acetate for up to five times without visible damage.

### Mechanical removal of epicuticular wax from LDs using gum arabic

Gum arabic (Sigma-Aldrich) was used as a third agent for the selective removal of epicuticular waxes. Because gum arabic is dissolved in water, potential solvent effects on wax extraction can be excluded (Jetter and Schäffer 2001). Before using gum arabic for analytical investigations, it was cleaned in hot chloroform for several hours to remove any organic contaminations. With gum arabic, epicuticular wax could only be stripped from LDs since the fragile CMs broke during gum arabic removal after drying. LDs were mounted in transpiration chambers like CMs, ensuring that the same surface area was treated every time. About 50 µl gum arabic dissolved in water (80 %) was applied to the upper side of the LDs with a brush. Drying time was about 60–90 min. After drying, gum arabic could only be removed in small, brittle pieces from the treated leaf area, which were then extracted in 4 ml chloroform for wax analysis. One and the same LD could be treated with gum arabic for up to five times without visible damage.

### Extraction of residual amounts of cuticular wax from CMs and LDs

After five treatments with either collodion or cellulose acetate, CMs were cut out of the transpiration chambers with a scalpel and the residual amount of cuticular wax remaining in CMs was fully extracted in chloroform (4 ml) over night at room temperature. After five treatments with gum arabic, LDs were cut out of the transpiration chambers with a scalpel and the CMs isolated enzymatically, since it has been shown that quantitative wax extraction with intact leaves can be incomplete (Riederer and Schneider 1989). The residual amount of cuticular wax remaining in the isolated CMs was fully extracted in chloroform (4 ml) over night at room temperature.

### Wax analysis using gas chromatography and mass spectrometry

Wax analysis was conducted as described in detail by Kurdyukov et al. (2006). All chloroform extracts of cuticular wax (epicuticular wax strips and residual wax) were spiked with an adequate amount of tetracosane (50 µl of a solution of 10 mg tetracosane in 50 ml chloroform; Fluka) serving as internal standard for wax quantification. Volumes of wax extracts were reduced to 500 µl under a gentle stream of nitrogen gas by evaporating the chloroform. Hydroxylic and carboxylic groups of alcohols and acids, being important constituents of *P. laurocerasus* wax, were transformed into the corresponding trimethylsilylethers and -esters. Derivatization was performed with *N,O*-bis(trimethylsilyl)-trifluoroacetamid (BSTFA; Machery-Nagel) and pyridine (Sigma Aldrich) for 40 min at 70 °C.

Samples of 1 µl were analysed by gas chromatography equipped with flame ionization detection (GC-FID; CG-Hewlett Packard 5890 series H) with on-column injection (30 m DB-1 i.d. 0.32 mm, film 0.2 µm; J&W Scientific). Wax compounds were identified by analyzing 1 µl samples by gas chromatography connected to mass spectrometry (GC–MS; quadrupole mass selective detector HP 5971, Hewlett-Packard). Identification of wax compounds was carried out by comparing the mass spectra obtained with mass spectra of known compounds stored in our in-house database.

### Scanning electron microscopy

Surfaces of *P. laurocerasus* LDs and CMs before and after selective removal of epicuticular wax were investigated by scanning electron microscopy (SEM; Leitz AMR 1000, Leitz). Samples of CMs and LDs were fixed to aluminium sample holders, freeze dried, sputtered (Polaron E5100, 15 mA, 30 s, Polaron Equipment LTD.) with a thin layer (18 nm) of gold and investigated at an accelerating voltage of 20 keV.

### Measurement of cuticular transpiration of CMs and LDs using ^3^H-labelled water

Cuticular transpiration of CMs and LDs of *P. laurocerasus* was measured using ^3^H-labelled water (^3^H_2_O; specific activity: 37 MBq g^−1^; Hartmann Analytik) as described in detail by Schreiber et al. (2001). CMs and LDs (*n* ≥ 10) were mounted on stainless steel transpiration chambers (Fig. 1a) filled with 800 µl radioactive donor solution of ^3^H_2_O (in the range of 10^13^ dpm m^−3^). The interfaces between the transpiration chambers and LDs or CMs were sealed with high-vacuum silicone grease (Wacker Chemie). The anatomically outer sides of the astomatous CMs and astomatous upper leaf sides of the LDs were facing the atmosphere, whereas the anatomically inner side of the CMs and the lower, stomatous side of the LDs were facing the radioactive donor solution. LDs were vacuum-infiltrated with radioactive donor solution and carefully blotted dry prior to mounting them on transpiration chambers. This ensured that ^3^H_2_O in the leaf was in equilibrium with the external donor solution of ^3^H_2_O (Schreiber 2001).

Transpiration chambers were closed with a special lid with a central open area of 1.13 cm^2^. This lid allowed tight connections between transpiration chambers (turned upside down) and scintillation vials (Fig. 1a). 250 µl glycerol (Sigma-Aldrich) was pipetted to the bottom of each scintillation vial. This ensured that all water, including all radioactive water that had diffused across the cuticle, was quantitatively trapped. Since the gas phase above pure glycerol has a relative humidity of 0 % (Slavik 1974), driving forces for cuticular transpiration were maximum and constant. Transpiration was measured at a constant temperature of 25º C. Before starting transpiration measurements, chambers were incubated overnight (25 °C, 0 % humidity) for 16 h. This allowed equilibration between the external gas phase in the scintillation vial and the cuticle and ensured that no traces of radioactivity were still adhering to the outer surface of LDs from infiltration with donor solution.

Transpiration of CMs and LDs was measured for 1–2 h (samples taken at 15 min, 30 min, 1 h and 2 h) before and after two consecutive treatments of the outer surfaces of CMs and LDs with collodion (15 µl), cellulose acetate (15 µl) or gum arabic (50 µl). In one experiment with CMs and collodion, transpiration was measured for up to 6 days with daily collodion treatments of the CMs. As negative control, LDs and CMs were always treated in the same way with the corresponding amounts of solvents (15 µl diethyl ether:ethanol, 15 µl acetone or 50 µl water). Since evaporation of the solvents diethyl ether:ethanol and acetone, with or without dissolved polymers, took <1–2 min, cuticular transpiration of CMs could be measured continuously without any significant interruption by the collodion or cellulose acetate treatments. Since evaporation of pure water or water with dissolved gum arabic took much longer, transpiration kinetics show breaks reflecting the drying time of water. At each sampling time, transpiration chambers were rapidly transferred to new scintillation vials containing pure glycerol. Glycerol in scintillation vials from the previous sampling, containing ^3^H-radiolabelled water that had diffused across the cuticle, was mixed with 5 ml scintillation cocktail (Ultima Gold, Perkin Elmer). Amounts of ^3^H_2_O in each vial were determined with a scintillation counter (LSA Tri-Carb 2800TR, Perkin Elmer) with an error of 2 %.

Plotting the amount of ^3^H_2_O diffused across the cuticle as a function of time resulted in individual transpiration kinetics for each CM or LD investigated. Linear regression lines were fitted to each transpiration curve and coefficients of determination of the regression lines (*r*^2^) were always better than 0.98. Effects of treating CMs and LDs with either collodion, cellulose acetate, gum arabic or the corresponding solvents were calculated by dividing the slopes of the regression lines after treatment by the slopes of the regression lines before treatment. Thus, values larger than 1 indicate an increase, values smaller than 1 indicate a decrease in transpiration, and values being statistically not different from 1 indicate no effect of the treatments on rates of cuticular transpiration. Permeances (P, in m s^−1^) were calculated by dividing the slopes of the regression lines (dpm s^−1^) by the exposed area (1.13 10^−4^ m^2^) and the driving force (10^13^ dpm m^−3^). Permeances are independent of all boundary conditions of the particular experiment (duration of the experiment, exposed cuticle area, driving force) and allow the comparison of cuticular transpiration rates obtained with different treatments (here wax removal), between different species and between different experiments. Even transpiration rates of synthetic polymers (e.g. polyethylene, polypropylene, PVC…) can be compared with those of cuticles (Riederer and Schreiber 2001).

### Statistics

Wax analysis was carried out with 5 combined replicates for each approach. In a first transport experiment (Fig. 6), transpiration kinetics of 10 CMs before and after 5 successive treatments with collodion were measured (collodion treatment and solvent control). In a second transpiration experiment (Fig. 7 showing three individual and representative CMs or LDs), transpiration kinetics of 7–10 CMs before and after two successive treatments with either collodion, cellulose acetate, gum arabic or the corresponding solvents were measured. Results of the wax analysis are given as means with standard deviations. Effects of polymer or solvent treatment (Fig. 8) were calculated from 7 to 10 CMs by dividing slopes of transpiration measurements before and after treatment. *T* tests were conducted to test whether measured effects of polymer or solvent treatment were significantly different from 1 (which would indicate there was no effect).

## Results

### Treatment of CMs and LDs with the different polymers

In a first set of preliminary experiments it was investigated if collodion, cellulose acetate or gum arabic can in principle be used for stripping epicuticular wax from CMs and LDs of *P. laurocerasus* mounted on transpiration chambers (Fig. 1) without causing visible damage. Up to five consecutive collodion or cellulose acetate treatments of mounted cuticles were possible in almost all cases without any visible indication that CMs were damaged. Only rarely and in no more than one out of ten experiments, CMs broke after the fourth or fifth strip or were partially pulled out of the slit between the transpiration chamber and the lid. Broken CMs and leaky chambers were discarded. With gum arabic only LDs could be used, because the polymer film could not be removed from isolated CMs without breaking them. This was due to the fact that the dried gum arabic adhered quite firmly to the cuticle and some force had to be applied for removing the brittle polymer film. This sometimes even caused damage to the surface of the intact leaf.

### Analysis of wax fractions obtained after repeated treatments of isolated *P. laurocersus* CMs with collodion

**Fig. 2:**
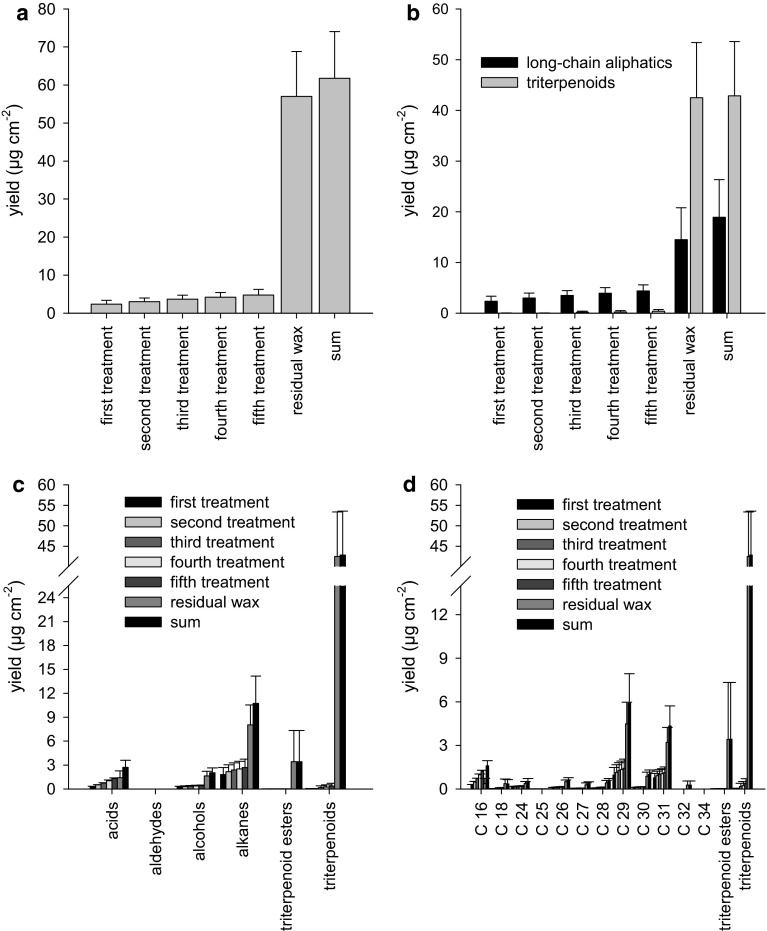
Composition of wax obtained with collodion strips from adaxial *P. laurocerasus* cuticles (CMs). **a** *First* to* fifth treatment* represent the five consecutive treatments of CMs with collodion for mechanically removing epicuticular wax, with the figures for the *second*, *third*, *fourth* and *fifth* *treatment* representing the wax amount obtained in the respective treatment plus the wax amounts obtained in all preceding treatments. Residual wax corresponds to the amount of wax still remaining in the CMs after selectively removing epicuticular wax in five treatments with collodion. *Sum* represents residual wax plus the wax removed in the five treatments. **b** Data of wax analysis plotted in the same way as in **a** but separately for linear, long-chain aliphatics and triterpenoids (including triterpenoid esters). It is clearly visible that linear, long-chain aliphatics are present in the epicuticular (*first* to* fifth treatment*) as well as in the intracuticular wax fraction (residual wax). Triterpenoids appear essentially just in the intracuticular wax fraction (residual wax); however, traces of triterpenoids start to appear in the third and consecutive treatments with collodion. **c** Data of wax analysis plotted in the same way as in **a** but grouped by substance class. Acids, aldehydes and alkanes are present in both the epi- (*first* to* fifth treatment*) and intracuticular wax fraction (residual wax), whereas alcohols, triterpenoid esters and triterpenoids are mostly or exclusively in the intracuticular wax fraction (residual wax). **d** Data of wax analysis plotted in the same way as in **a** but arranged according to the different chain lengths of the wax compounds

The total wax amount of *P. laurocerasus*, calculated from the collodion strips and the residual amount, was 61.8 ± 12.2 µg cm^−2^ (Fig. 2a, right bar, sum). *P. laurocerasus* wax was composed of linear, long-chain aliphatic molecules (acids, aldehydes, alcohols, alkanes and esters) and pentacyclic triterpenoids (ursolic acid, oleanolic acid and uvaol), the latter representing about 70 % (42.9 ± 10.7 µg cm^−2^) of the total wax amount (Fig. 2b, black and grey right bars, sum). Alkanes and acids represented the predominant substance classes of the linear, long-chain aliphatics (Fig. 2c). Chain lengths of linear, long-chain aliphatics ranged from C_16_ to C_34_ (Fig. 2d). Comparable results were obtained with collodion and LDs (data not shown).

Each of the five consecutive strips of collodion was separately extracted in chloroform and the wax amount determined. Results are presented as the sum of wax obtained from the respective collodion strip plus wax amounts obtained from all preceding strips (Fig. 2a). The largest amounts of wax (2.4 ± 0.9 µg cm^−2^) were obtained with the first collodion treatment (Fig. 2a, first treatment). A weak and statistically non-significant increase of wax could be observed in the four subsequent collodion treatments (Fig. 2a, second to fifth treatment) leading to a final wax amount of 4.8 ± 1.5 µg cm^−2^. After five treatments the collodion-treated areas of the CMs were cut out of the chamber with a scalpel and the residual wax amounts after stripping were determined by extraction in chloroform (Fig. 2a, residual wax). The residual amount of wax was 57.0 ± 11.8 µg cm^−2^, which indicates that the bulk of the waxes could not be removed with collodion.

Only the long-chain aliphatics were obtained in significant amounts with the five collodion treatments (Fig. 2b). Beginning with the third treatment, small traces of triterpenoids appeared (Fig. 2b). However, the bulk of the triterpenoids (including triterpenoid esters) and about two-thirds of the long-chain aliphatics were located in the residual wax fraction (Fig. 2b). It was mostly alkanes (1.8 ± 0.8 µg cm^−2^) and acids (0.3 ± 0.4 µg cm^−2^) that were removed from the CM surface with collodion (Fig. 2c). The amounts of acids increased with each treatment, whereas alkanes reached a plateau. Triterpenoid esters and triterpenoids appeared almost exclusively in the residual wax fraction. Substances with shorter chain lengths (especially C_16_ acid and C_18_ acid) were largely removed from the CM surface with the collodion treatments (Fig. 2d). Substances with higher chain lengths appeared in significant amounts in the residual wax fraction and triterpenoids and triterpenoid esters appeared almost exclusively in the residual wax fraction (Fig. 2d).

### Analysis of wax fractions obtained after repeated treatments of isolated *P. laurocersus* CMs with cellulose acetate

**Fig. 3:**
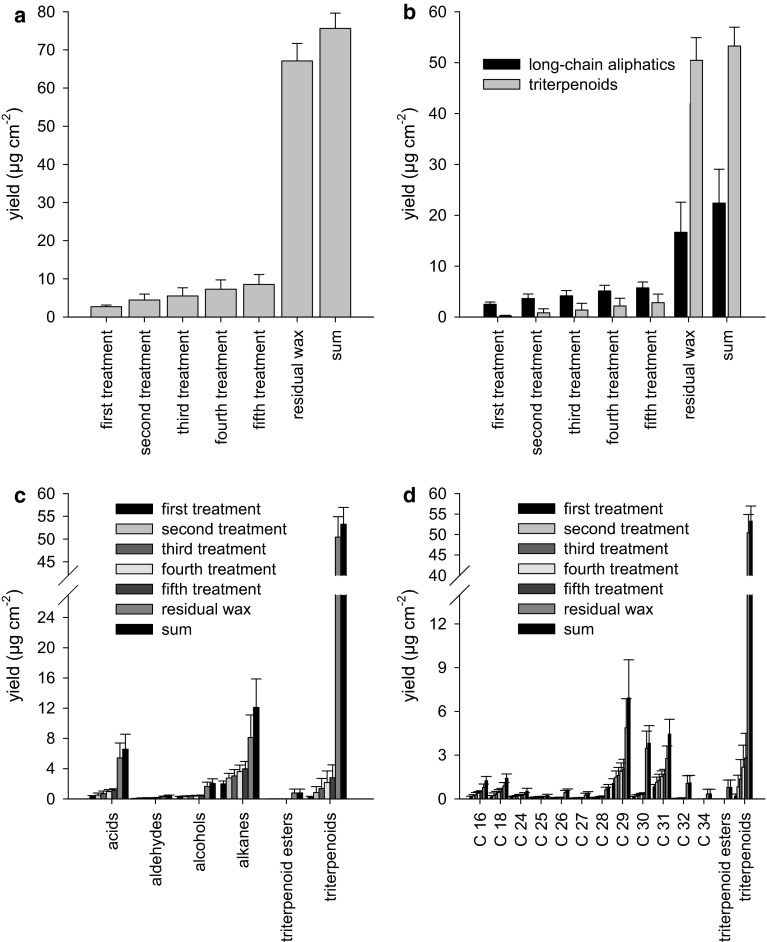
Composition of wax obtained with cellulose acetate strips from adaxial *P. laurocerasus* cuticles (CMs). **a** *First* to* fifth treatment* represent the five consecutive treatments of CMs with cellulose acetate for mechanically removing epicuticular wax, with the figures for the *second*, *third*, *fourth* and *fifth* *treatment* representing the wax amount obtained in the respective treatment plus the wax amounts obtained in all preceding treatments. Residual wax corresponds to the amount of wax still remaining in the CMs after selectively removing epicuticular wax in five treatments with cellulose acetate. Sum represents residual wax plus the wax removed in the five treatments. **b** Data of wax analysis plotted in the same way as in **a** but separately for linear, long-chain aliphatics and triterpenoids (including triterpenoid esters). It is clearly visible that linear, long-chain aliphatics are present in the epicuticular (first to fifth treatment) as well as in the intracuticular wax fraction (residual wax). The bulk of the triterpenoids is in the intracuticular wax fraction (residual wax), but considerable amounts of triterpenoids already appear in the first and consecutive treatments with cellulose acetate. **c** Data of wax analysis plotted in the same way as in **a** but grouped by substance class. Acids, aldehydes and alkanes are present in both the epi- (*first* to* fifth treatment*) and intracuticular wax fraction (residual wax), whereas alcohols, triterpenoid esters and triterpenoids are mostly or exclusively in the intracuticular wax fraction (residual wax). **d** Data of wax analysis plotted in the same way as in **a** but arranged according to the different chain lengths of the wax compounds

The preceding experiment of removing epicuticular wax with consecutive strips was repeated in exactly the same way using cellulose acetate. The largest wax amounts (2.7 ± 0.4 µg cm^−2^) were again obtained with the first strip (Fig. 3a, first treatment). An increase in the total amount of wax removed by stripping could be observed in the four subsequent cellulose acetate strips (Fig. 3a) leading to a final wax amount of 8.5 ± 2.6 µg cm^−2^ (sum of all 5 strips). The residual wax amount after stripping was 67.1 ± 4.6 µg cm^−2^ (Fig. 3a, residual wax), indicating that the majority of the wax could not be removed with cellulose acetate. The sum of residual wax plus the wax obtained from all cellulose acetate strips was 75.6 ± 4.73 µg cm^−2^ (Fig. 3a).

Mostly long-chain aliphatics but also a small amount of triterpenoids could be removed from the surface of isolated *P. laurocerasus* CMs with the very first strip (Fig. 3b). However, the largest amount of triterpenoids (including triterpenoid esters) was detected in the residual wax fraction (Fig. 3b). Cellulose acetate removed mostly alkanes (2.0 ± 0.4 µg cm^−2^) from the CM surface (Fig. 3c). Most of the triterpenoid esters and triterpenoids appeared in the residual wax fraction. The chain length distributions did not indicate that cellulose acetate stripping discriminated between shorter and longer chain lengths (Fig. 3d).

### Analysis of wax fractions obtained after repeated treatments of intact *P. laurocersus* LDs with gum arabic

**Fig. 4:**
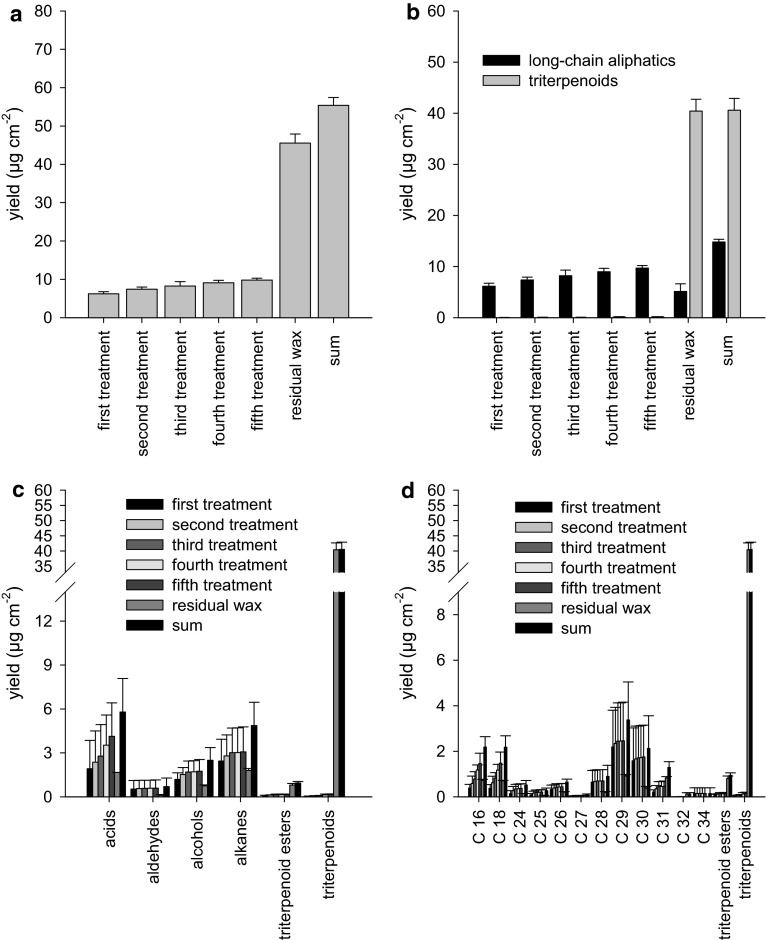
Composition of wax obtained with gum arabic strips from adaxial *P. laurocerasus* cuticles (CMs). **a** *First* to* fifth treatment* represent the five consecutive treatments of LDs with gum arabic for mechanically removing epicuticular wax, with the figures for the second, third, fourth and fifth treatment representing the wax amount obtained in the respective treatment plus the wax amounts obtained in all preceding treatments. Residual wax corresponds to the amount of wax still remaining in the LDs after selectively removing epicuticular wax in five treatments with gum arabic and enzymatically isolating the cuticles. Sum represents residual wax plus the wax removed in the five treatments. **b** Data of wax analysis plotted in the same way as in **a** but separately for linear, long-chain aliphatics and triterpenoids (including triterpenoid esters). It is clearly visible that linear, long-chain aliphatics are present in the epicuticular (*first* to* fifth treatment*) as well as in the intracuticular wax fraction (residual wax). Triterpenoids are essentially in the intracuticular wax fraction (residual wax); however, traces of triterpenoids start to appear in the third and consecutive treatments with gum arabic. **c** Data of wax analysis plotted in the same way as in **a** but grouped according to the substance class. Acids, aldehydes, alcohols and alkanes are present in both the epi- (*first* to* fifth treatment*) and intracuticular wax fraction (residual wax), whereas triterpenoid esters and triterpenoids are mostly or exclusively in the intracuticular wax fraction (residual wax). **d** Data of wax analysis plotted in the same way as in **a** but arranged according to the different chain lengths of the wax compounds

In a third approach, gum arabic was used for mechanically removing epicuticular wax from LDs (Fig. 4). The largest amounts of wax (6.2 ± 0.5 µg cm^−2^) were again obtained from the first strip (Fig. 4a, first treatment). A slight increase of total wax removed could be observed in the four subsequent gum arabic strips, leading to a final wax amount of 9.8 ± 0.5 µg cm^−2^ (Fig. 4a). Treated areas of the LDs were cut out of the chambers using a scalpel and residual wax amounts were determined after enzymatically isolating the cuticles and extracting the membranes in chloroform (Fig. 4a, residual wax). The residual amount of wax was 45.6 ± 2.3 µg cm^−2^, indicating again that the bulk of the wax could not be removed by treating the surface with gum arabic. The sum of the residual wax plus the wax obtained with gum arabic treatments was 55.4 ± 2.0 µg cm^−2^ (Fig. 4a).

A large fraction of the long-chain aliphatics could be removed from the surface of the intact *P. laurocerasus* LDs using gum arabic (Fig. 4b), but the amount of triterpenoids removed was extremely low (0.04 ± 0.01 µg cm^−2^) albeit already measurable in the first strip. However, the largest amount of triterpenoids (including triterpenoid esters) was located in the residual wax fraction (Fig. 4b). When plotting wax amounts arranged by substance class (Fig. 4c), it can be seen that mostly acids (2.0 ± 0.4 µg cm^−2^), alcohols (1.18 ± 0.45 µg cm^−2^) and alkanes (2.44 ± 1.49 µg cm^−2^) were removed from the LD surface with gum arabic. From the remaining substance classes, especially triterpenoid esters and triterpenoids appeared in the residual wax fraction (Fig. 4c). Chain length distribution did not indicate discrimination between shorter and longer chain lengths by gum arabic stripping (Fig. 4d).

### Scanning electron microscopic investigation for verifying epicuticular wax removal

**Fig. 5:**
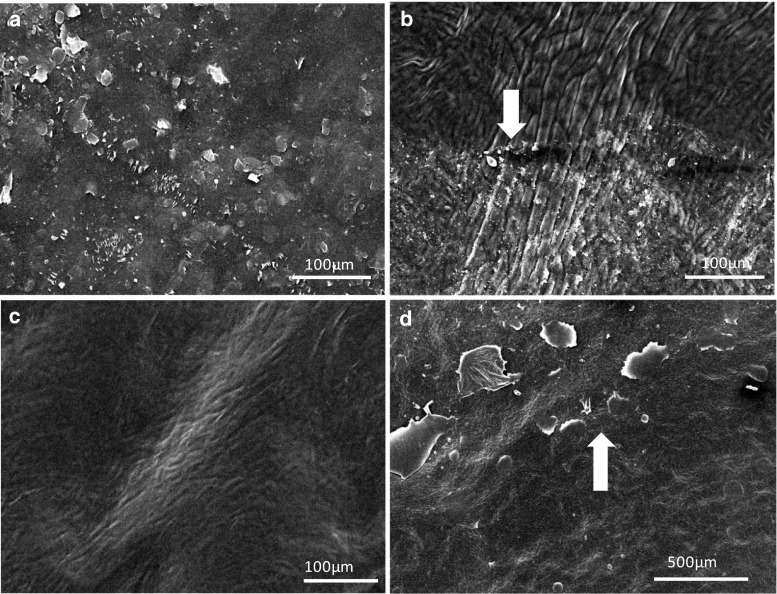
Scanning electron microscopic pictures of the anatomical outer side of cuticles (CMs) isolated from the upper astomatous leaf side of *P. laurocerasus* leaves and LDs. **a** Outer surface of an untreated *P. laurocerasus* CM showing the occurrence of epicuticular wax platelets. **b** Outer surface of a *P. laurocerasus* CM treated twice with collodion only on the upper half, clearly showing the border (*arrow*) between the treated and untreated halves. **c** Outer surface of a *P. laurocerasus* CM after two consecutive treatments with cellulose acetate. The surface appears smooth and all wax platelets have disappeared. **d** Outer surface of a *P. laurocerasus* LD treated twice with gum arabic on the lower half. A weak border (*arrow*) appears between the untreated and treated halves of the leaf disc and the treated area appears smooth

In addition to the chemical analytical approach, we used scanning electron microscopy for studying the effect of collodion, cellulose acetate and gum arabic stripping on cuticle surface micromorphology of *P. laurocerasus* CMs and LDs. Untreated CMs (Fig. 5a) and LDs had a rough appearance, showing a loose and irregular, but distinct coverage with epicuticular wax platelets. When collodion was carefully applied twice on one half of the CM (Fig. 5b, upper half), leaving the other half untreated (Fig. 5b, lower half), a clear border between treated and untreated halves of the CM was visible (Fig. 5b, white arrow). Most epicuticular wax platelets disappeared already after treating the surface of CMs once with collodion (not shown) and epicuticular wax platelets completely disappeared after treating the CM surface twice with collodion, at which point CMs exhibited a very smooth and clean appearance (Fig. 5b, upper half). Treating the isolated *P. laurocerasus* CM twice with cellulose acetate also led to a smooth appearance of the surface free of epicuticular wax platelets (Fig. 5c), again indicating the successful removal of epicuticular waxes. With gum arabic successful wax removal could be visualised using intact LDs (Fig. 5d). After two consecutive treatments the surface appeared smooth (Fig. 5d, lower half) and free of wax platelets. In comparison with the collodion treatment, a weaker but still significant border between untreated and treated regions on the LD appeared (Fig. 5d, white arrow).

### Measurement of cuticular transpiration using ^3^H-labelled water

**Fig. 6:**
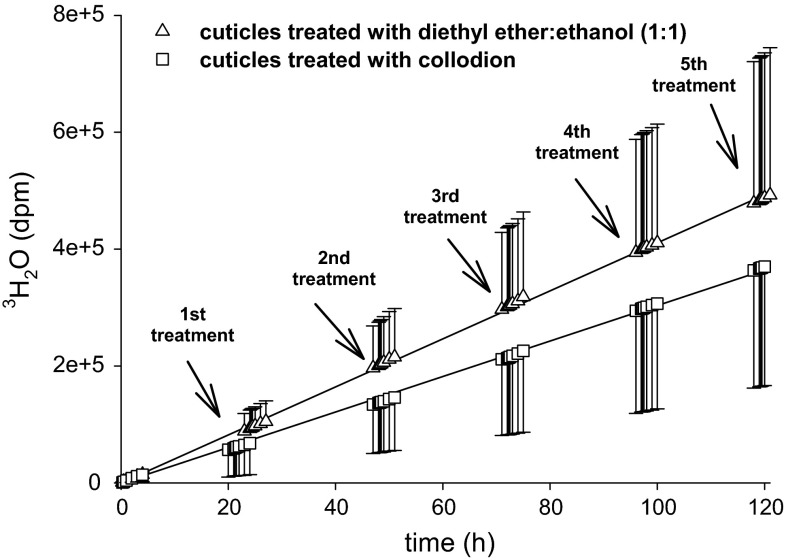
Diffusion of ^3^H-labelled water across isolated cuticles (CMs) of *P. laurocerasus* before and after five consecutive treatments (*black arrows*) with collodion (*squares*) or diethyl ether:ethanol (*triangles*). During transpiration measurements the outer cuticle surface was either stripped with a thin layer of collodion, which was removed 30–60 s after solvent evaporation, or treated with 15 µl solvent (diethyl ether:ethanol; 1.1, v/v; *black arrow*), which was allowed to evaporate. Regression lines were fitted to the transpiration kinetics. Coefficients of determination *r* ^2^ were better than 0.99

**Fig. 7:**
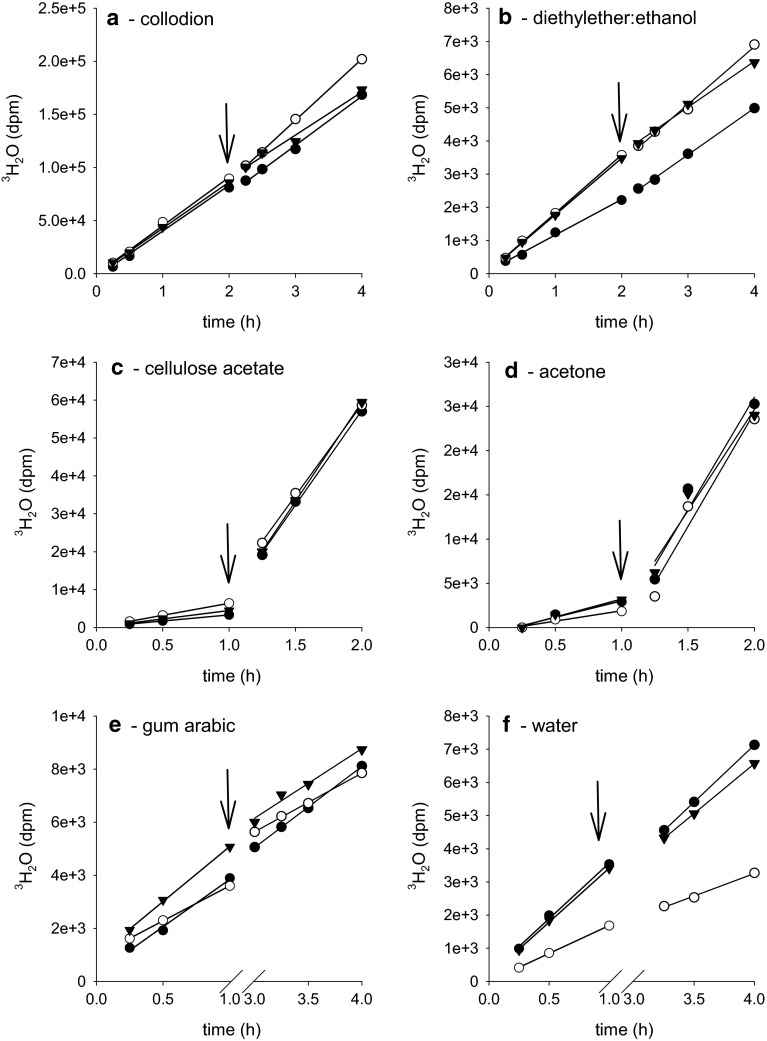
Diffusion of ^3^H-labelled water across isolated cuticles (CMs) of *P. laurocerasus* and across the cuticles of intact leaf disks (LDs) before and after treatment (*black arrows*) with collodion, cellulose acetate, gum arabic or corresponding solvents. **a** Transpiration kinetics (amounts of ^3^H-labelled water having crossed the cuticle, as a function of time) of three representative *P. laurocerasus* CMs before and after treating them twice with collodion (*black arrow*). **b** Transpiration kinetics of three *P. laurocerasus* CMs before and after treating them twice with 15 µl solvent (diethyl ether:ethanol; 1.1, v/v; *black arrow*). **c** Transpiration kinetics of three examples of *P. laurocerasus* CMs before and after treating the surface with cellulose acetate (*black arrow*). **d** Transpiration kinetics of *P. laurocerasus* CMs before and after treating them twice with 15 µl acetone (*black arrow*). **e** Transpiration kinetics of *P. laurocerasus* LDs before and after treating them with gum arabic (*black arrow*). **f** Transpiration kinetics of *P. laurocerasus* CMs before and after treating them twice with 15 µl water (*black* *arrow*). Regression lines were fitted to the kinetics before and after the different treatments. Coefficients of determination *r* ^2^ were always better than 0.98

In order to test whether removal of epicuticular wax had an effect on cuticular barrier properties, diffusion of ^3^H-labelled water across isolated CMs and cuticles of intact LDs mounted on transpiration chambers (Fig. 1a) was measured. In the first experiment, CMs were treated up to five times with collodion or diethyl ether:ethanol (Fig. 6). Slopes of the transpiration kinetics measured before and after the treatments did not change, which indicated that rates of cuticular transpiration were not affected by treatments with collodion or the corresponding solvent at all (Fig. 6). Since the microscopic investigation showed that two treatments with the three different polymers were sufficient for removing essentially all epicuticular wax crystalloids (Fig. 5), in the following experiments CMs and LDs were treated only twice (Fig. 7). After two consecutive collodion strips of CMs (Fig. 7a, arrows) and gum arabic strips of LDs (Fig. 7e, arrows), transpiration rates did not significantly increase (Fig. 7a, e). Identical results were obtained when treating CMs twice with diethyl ether:ethanol (Fig. 7b) or LDs with water (Fig. 7f). However, transpiration rates significantly increased after two consecutive strips of CMs with cellulose acetate (Fig. 7c) or after two treatments with acetone (Fig. 7d).

**Fig. 8:**
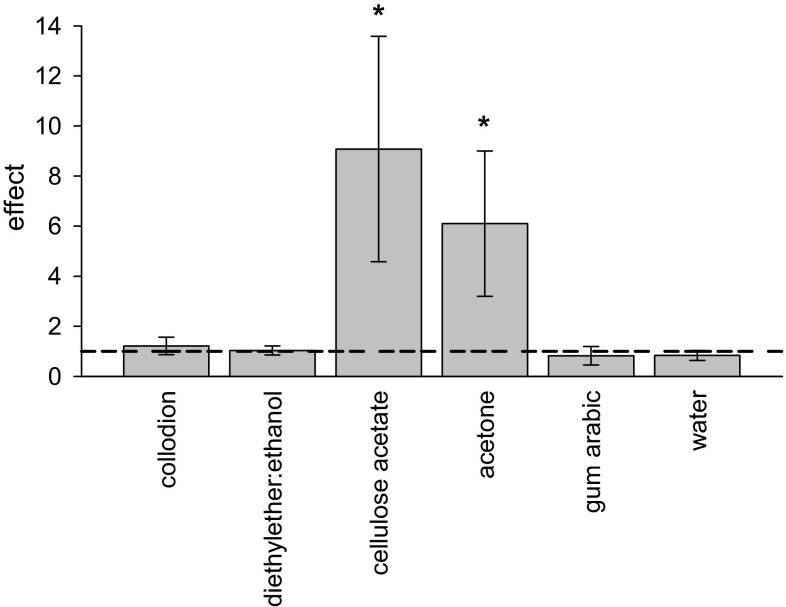
Effects on cuticular transpiration of *P. laurocerasus* after treatment with collodion, cellulose acetate, gum arabic or corresponding solvents (diethyl ether:ethanol, acetone or water). The outer surfaces of astomatous cuticles (CM) isolated from the upper leaf side of *P. laurocerasus* were treated twice with either collodion or cellulose acetate for selective removal of the epicuticular wax. As negative controls CMs were treated twice with either diethyl ether:ethanol or acetone. The upper surface of intact leaf disks (LD) of *P. laurocerasus* was treated twice with either gum arabic for selective removal of epicuticular wax or twice with water. Effects were calculated by dividing the regression coefficients of cuticular transpiration (diffusion of ^3^H-labelled water across cuticles) after treatment with the polymers or solvents by the regression coefficients of cuticular transpiration before treatment. Effects of cellulose acetate or acetone treatments of CMs were significantly different from 1 (*asterisks*)

The effects of the treatments, calculated by dividing the slopes of regression lines fitted to the transpiration kinetics after stripping by the slopes of the regression lines before stripping, varied between 0.92 and 1.90 for collodion, between 4.47 and 15.58 for cellulose acetate, and between 0.43 and 1.97 for gum arabic. Effects of solvent treatments varied between 0.81 and 1.29 for CMs treated with diethyl ether:ethanol, between 2.31 and 10.59 for isolated *P. laurocerasus* CMs treated with acetone, and between 0.59 and 1.02 for CMs treated with water. The mean effects of the treatments with collodion, gum arabic and the respective solvents were statistically not significantly different from 1 (Fig. 8). In the case of cellulose acetate and acetone, the calculated effects were statistically different from 1.

## Discussion

### Mechanical removal of epicuticular wax with collodion, cellulose acetate or gum arabic

For our experimental approach we were searching for a fast and safe method allowing a rapid and efficient removal of epicuticular wax from CMs. Simultaneously, it had to be ensured that fragile CMs were not affected or damaged by this treatment. Since cuticular wax is soluble in organic solvents, it has been argued that selective removal of epicuticular wax should be carried out without organic solvents and using water based polymers instead (Ensikat et al. 2000; Jetter et al. 2000; Jetter and Schäffer 2001; Neinhuis et al. 2001; Koch et al. 2004). We first tried the different “solvent-free” methods suggested for mechanical wax removal (freezing of liquid glycerol or water, gum arabic, commercially available water based glue); however, none of them could be applied to CMs, because the membranes regularly broke or were severely damaged, which rendered them unusable for the transpiration measurements. Therefore, we reinvestigated other methods (collodion and cellulose acetate) based on organic solvents (diethyl ether:ethanol and acetone) and compared them to gum arabic.

Using gum arabic for selective removal of epicuticular wax, Jetter and Schäffer (2001) described for the first time the arrangement of cuticular wax of *P. laurocerasus* in chemically distinct layers, with triterpenoids being exclusively located in the intracuticular wax fraction. Thus, the appearance of triterpenoids in larger amounts in mechanical strips indicates that intracuticular wax must have been removed from the cuticle. When using collodion, considerable amounts of triterpenoids (in the range of 3–5 µg cm^−2^) appeared in the wax that was mechanically removed from the leaf surface (Jetter et al. 2000). From this, the authors concluded that the organic solvent dissolves and mobilises intracuticular triterpenoids after application of collodion to the leaf surface, and therefore triterpenoids were detected in the polymer film after solvent evaporation. Thus, they suggested avoiding the use of collodion for epicuticular wax removal, since it cannot be excluded that intracuticular wax fractions are mobilised.

When using cellulose acetate dissolved in acetone for epicuticular wax removal, in fact considerable amounts of triterpenoids (in the range of 0.2–3 µg cm^−2^) were detected in the present study (Fig. 3b). Acetone acted as a solvent, dissolving intracuticular wax as predicted by Jetter et al. (2000), and should therefore be avoided in further experiments. However, when comparing collodion dissolved in diethyl ether:ethanol (Fig. 2) to gum arabic dissolved in water (Fig. 4), we were able to detect similar trace amounts of triterpenoids (between 0.02 and 0.2 µg cm^−2^) in epicuticular wax fractions obtained with collodion from CMs (Fig. 2b) or with gum arabic from LDs (Fig. 4b). Compared with the total amounts of intracuticular triterpenoids (about 42 µg cm^−2^), the amounts of triterpenoids detected here in the collodion and gum arabic strips were in the range of a few per mill. In our experiments, diethyl ether:ethanol obviously did not dissolve triterpenoids in significant amounts since identical, minute amounts of triterpenoids were found with collodion (Fig. 2b) and gum arabic (Fig. 4b). In our very first experiments we found in fact that by dipping isolated cuticles for only a few seconds in acetone, ten times more triterpenoids were extracted than with diethyl ether:ethanol (data not shown). This clearly indicated that acetone interacted more effectively or for longer (due to slower evaporation) than diethyl ether:ethanol with the intracuticular wax fraction.

Possible reasons why we obtained different results with collodion compared to Jetter et al. (2000) might be related to the fact that we used collodion from a different supplier (Fluka instead of Merck), that it had a different concentration (4–8 % nitrocellulose instead of 4 %) and that it was dissolved at a 1:1 (v/v) ratio in diethyl ether:ethanol and not at a 3:1 (v/v) ratio (Jetter et al. 2000). Thus, from our results it is justified to conclude that collodion represents a suitable technique for selectively removing epicuticular wax from *P. laurocerasus* CMs, allowing the measurement of cuticular transpiration before and directly after wax removal. Since it was observed (for other species, not for *P. laurocerasus*) that epicuticular wax regeneration can start within minutes after mechanical wax removal (Koch et al. 2004), it is important that this technique is also fast enough for measuring cuticular transpiration just one to 2 min after wax removal, which is not possible with gum arabic due to the slow evaporation of water. Collodion offers the advantages that it can be applied rapidly to the CM surface, evaporation of the solvent and formation of the polymer film is fast (within 30–60 s), and it does not damage CMs.

### Chemical composition of cuticular wax of *P. laurocerasus* after treatment with collodion, cellulose acetate or gum arabic

A detailed wax analysis was carried out in order to verify further that our approach of using collodion instead of gum arabic was appropriate for selectively removing epicuticular wax and that results were similar to those obtained with gum arabic (Jetter and Schäffer 2001). Their observation that the epicuticular wax fraction is exclusively composed of linear, long-chain aliphatic molecules could essentially be confirmed here (Figs. 2b, 4b). Looking at the wax data more closely, it is evident that there is a steady, although not statistically significant, increase of wax from the first to the fifth strip. In addition, it can be seen that amounts of triterpenoids increased, although only in traces, with the third treatment of the surface of the CM (Fig. 2b). Therefore, for the selective removal of epicuticular wax for the transpiration experiments CMs were treated only twice with collodion (Fig. 5).

The results achieved with gum arabic (Fig. 4) were similar to the results obtained with collodion (Fig. 2) and they confirmed published results obtained with gum arabic (Jetter and Schäffer 2001). With the first strip, mostly long-chain aliphatics could be removed from the surface of intact leaf disks (Fig. 4). Triterpenoids were again found only in traces, indicating that essentially no waxes were mobilised from the inner part of the cuticle with gum arabic. The amounts of cuticular wax obtained from mechanical removal of epicuticular wax were higher in the gum arabic treatments (6.2 µg cm^−2^ from the first strip, 9.8 µg cm^−2^ from all five strips together) compared with the collodion treatments (2.4 and 4.7 µg cm^−2^, respectively). Total amounts of cuticular wax did not differ between collodion (61.2 µg cm^−2^) and gum arabic (55.4 µg cm^−2^). One explanation for these differences might be the fact that experiments with CMs and LDs were carried out with plant material harvested in different years. Our experiments started in 2009 with CMs isolated in 2008 from fully grown *P. laurocerasus* leaves. Experiments with LDs were conducted with fully grown leaves harvested in 2012 and at a different time of the year than in 2008. It has been shown that cuticular wax can undergo pronounced quantitative as well as qualitative seasonal variation without necessarily affecting rates of cuticular transpiration (Hauke and Schreiber 1998).

Quantitative differences became evident when comparing the total amounts of wax determined for the CMs and LDs in this study (between 55.4 and 75.6 µg cm^−2^) to published values (28 µg cm^−2^; Jetter et al. 2000). Several reasons could account for this difference. For total wax analysis we extracted cuticular wax over night from isolated CMs, whereas wax extraction by Jetter et al. (2000) was done in 30 s with LDs. It has been reported for *Citrus aurantium* (Riederer and Schneider 1989) that quantitative wax extraction was only possible with isolated CMs. It must also be kept in mind that in the two studies most probably different cultivars of *P. laurocerasus* were used, not necessarily belonging to the same genotype.

### Scanning electron microscopy investigation for verifying epicuticular wax removal

Scanning electron microscopic observations were carried out independently, confirming the analytical results (Figs. 2, 3, 4), which indicated the successful removal of epicuticular wax from *P. laurocersus* leaf surfaces with collodion, cellulose acetate and gum arabic. The irregular but distinct epicuticular wax platelets (Fig. 5a) could be removed completely from the surface of CMs and LDs with two consecutive treatments. The clear difference between the rough and smooth surface of the CM before and after treatment can best be seen when only half of the CM was treated with collodion or gum arabic (Fig. 5b, d). Treating the surface of isolated *P. laurocerasus* CMs twice with cellulose acetate also led to a smooth appearance of the membrane (Fig. 5c). Thus, from our analytical and microscopical results we concluded that two subsequent treatments of the *P. laurocerasus* leaf surface are sufficient for selectively removing epicuticular wax (Fig. 5).

### Effects of epicuticular wax removal on cuticular transpiration using ^3^H-labelled water

Cuticular transpiration has been measured for isolated cuticles of a large number of species (Schreiber and Riederer 1996; Riederer and Schreiber 2001) and for intact leaf disks collected from a smaller number of species (Schreiber 2001). In a direct comparison of CMs and LDs it could be shown that rates of cuticular transpiration (Schreiber et al. 2001) as well as solute permeability (Kirsch et al. 1997) were not affected by isolation of the CM. Mean published values of P for *P. laurocerasus* ranged from 1.0 × 10^−10^ to 9.0 × 10^−10^ m s^−1^ (Schreiber and Riederer 1996; Schreiber et al. 2001; Schreiber 2002), depending on the batch of isolated cuticles used in different experiments conducted in different years and series. Mean values of P calculated here from the transpiration rates in the various experimental setups (Fig. 7) showed a similar variation in the same range (1.5 × 10^−10^–1.0 × 10^−9^ m s^−1^). This was due to the fact that some of the different experimental series (Fig. 7a–f) were conducted using different batches of cuticles, isolated in consecutive years. Total wax extraction, however, led to an increase of cuticular permeabilities for water (Schönherr 1976; Schönherr and Lendzian 1981) and solutes (Riederer and Schönherr 1985) by two to three orders of magnitude. A similar effect of a 94-fold increase of P upon wax extraction was described for *P. laurocerasus* CMs (Schreiber 2002).

Selective removal of epicuticular wax from the surface of *P. laurocerasus* CMs and LDs with collodion (Fig. 7a) or gum arabic (Fig. 7e) did not increase transpiration, as mean effects were statistically not different from 1 (Fig. 8). By contrast with the treatments using collodion (Fig. 7a), gum arabic (Fig. 7e), diethyl ether:ethanol (Fig. 7b) and water (Fig. 7f), the treatments with cellulose acetate (Fig. 7c) or acetone alone (Fig. 7d) significantly increased P (Fig. 8). As acetone dissolves significant amounts of intracuticular triterpenoids (Fig. 3), it can be concluded that cellulose acetate dissolved in acetone cannot be used for answering the question to what extent epicuticular wax contributes to the formation of the cuticular transpiration barrier. Evidently, acetone significantly damages the cuticular transpiration barrier (Fig. 7c, d).

However, with the collodion and gum arabic treatments only epicuticular waxes were removed. Since rates of cuticular transpiration did not change after two (Fig. 7) or even after five consecutive collodion treatments (Fig. 6), it can be concluded that the transpiration barrier of the *P. laurocerasus* cuticle is predominantly established by the intracuticular wax fraction. This conclusion is supported by the observation that partially dissolving intracuticular triterpenoids by treatment with acetone alone, or with cellulose acetate dissolved in acetone, leads to a significant, 5–10-fold increase in *P* (Fig. 7). Significant effects on P after removal of epicuticular wax have been described for tomato (Vogg et al. 2004) and cherry fruit cuticles (Knoche et al. 2000). However, fruit cuticles typically have a much higher water permeability than leaf cuticles (Becker et al. 1986). Thus, it can be speculated that the contribution of the epicuticular wax layer to the overall resistance of leaf cuticles is not noticeable because the resistance of the underlying cuticle is so high that it renders the epicuticular resistance insignificant, whereas this is not the case with highly permeable fruit cuticles. To test this hypothesis, the relative effects of epicuticular wax stripping on P in a much larger number of leaf and fruit cuticles, covering a broad range of cuticular permeabilities, would need to be investigated.

Why is it ecologically relevant whether epicuticular wax contributes to the cuticular transpiration barrier? It has been established that neither the thickness of the CM itself (Kamp 1930) nor the thickness or amount of cuticular wax (Becker et al. 1986; Schreiber and Riederer 1996) correlates with P. However, there is good evidence that the molecular arrangement of cuticular wax, especially the degree of crystallinity or the ratio between crystalline and amorphous wax regions, determines the cuticular transport barrier (Schreiber et al. 1997; Merk et al. 1998). Model calculations, based on measuring transpiration of water across monomolecular layers formed by linear, long-chain aliphatic molecules (Archer and La Mer 1955), led to the conclusion that a single monolayer composed of linear, long-chain aliphatic wax molecules with average chain lengths between C_30_ and C_40_ would be sufficient for establishing the observed values of *P* (Schreiber and Schönherr 2009). A monolayer composed of wax molecules with a chain length between C_30_ and C_40_, corresponding to chain lengths often found for cuticular wax (Samuels et al. 2008), would be 4–5 nm thick. This would also correspond to the thickness of 3–5 nm of a monolayer found forming on the cuticle surface after mechanical wax removal (Koch et al. 2004).

Thus, in view of this hypothetical “monolayer model” of the cuticular transpiration barrier, the observation that exclusively linear, long-chain aliphatic wax molecules occur on the outer surface of the *P. laurocerasus* CM was very exciting (Jetter et al. 2000). This could have represented a further piece of evidence supporting this “monolayer model”, because it would be conceivable that linear, long-chain aliphatic wax molecules could form such highly organised monolayers on the cuticle surface and thus establish the transpiration barrier of the CM. In fact, very small amounts of epicuticular wax, in the range of 1–2 µg cm^−2^, would be sufficient for establishing a monolayer of this thickness (Schreiber and Schönherr 2009).

However, our transpiration experiments clearly contradict this model, at least for *P. laurocerasus*, since removal of epicuticular wax with collodion (Fig. 7a) and gum arabic (Fig. 7e) did not increase *P*. There are, in fact, more reasons to argue against this “monolayer model”. Leaf surfaces are permanently interacting with abiotic (wind, rain, ice, dust…) and biotic environmental factors (insects, fungi and other microorganisms…). This would lead to permanent disturbances and damage of such a fragile, only 4–5 nm thin, monolayer of wax molecules on the outer surface of the CM. Biologically it makes a lot more sense to establish the cuticular transpiration barrier, which is vital for protecting plants from uncontrolled water loss, within the mechanically stable cutin polymer and thus protecting the barrier from these potentially damaging environmental influences.

Even though the highly ordered and layered epicuticular wax is not relevant for the formation of the cuticular transpiration barrier of *P. laurocerasus*, there could be important reasons for this wax arrangement giving the leaf surface its very individual chemical “taste”. It could be relevant for many other aspects of the plant/environment interaction, e.g. repelling potentially feeding insects or attracting beneficial epiphyllic fungi and other microorganisms. Up to now, the experimental method used here has only been employed for investigating *P. laurocerasus*. Therefore, further experiments with additional species are needed before more general conclusion can be drawn. Our approach of measuring cuticular transpiration before and again directly after removal of epicuticular wax also works with disks cut from intact leaves. This allows investigating species that have astomatous upper leaf sides but where cuticle isolation is not possible. This significantly increases the number of species that can potentially be investigated. Thus, future experiments using this combined approach of measuring transpiration in parallel to wax analysis will give further insights into structure and function of epicuticular wax, the formation of the cuticular transpiration barrier and plant/environment interactions occurring across the cutinised leaf surface.

#### *Author contribution statement*

LS conceived and designed the experiments. VZ performed the experiments. VZ and LS analysed the data. LS and VZ wrote the manuscript.

## Abbreviations

CM: Cuticular membrane
LD: Leaf disk
P: Permeance
